# IN-HOSPITAL MORTALITY AMONG DEMENTIA PATIENTS IN CHINA: EVIDENCE FROM HOSPITAL ADMINISTRATIVE DATA

**DOI:** 10.1093/geroni/igae098.3080

**Published:** 2024-12-31

**Authors:** Zhuoer Lin, Fang Ba, Heather Allore, Xi Chen

**Affiliations:** University of Illinois Chicago, Chicago, Illinois, United States; Yale University, New Haven, Connecticut, United States; Yale University, New Haven, Connecticut, United States; Yale University, New Haven, Connecticut, United States

## Abstract

Despite the fast-growing dementia patient population, dementia care in China is still inadequate and inequitable that may lead to undesirable disease outcomes. We assessed the in-hospital mortality for dementia patients in China and its variations across patient and hospital characteristics. Inpatient medical records of Chinese older adults admitted to 1,917 hospitals during 2017-2019 with a primary diagnosis of dementia were analyzed; and multivariate logistic regression was used to assess the association of in-hospital mortality with age bands, sex, working status, insurance type, major comorbidity, admission type, and hospital level. Out of 51,525 included admissions, 363 dementia patients died in a hospital (0.70%, 95% CI, 0.63% - 0.78%). The odds of in-hospital mortality were significantly higher for patients aged 80 and over, having lung diseases, and for those admitted through emergency room or hospital transfers, or those admitted to lower-level hospitals compared to their higher-level counterparts. These findings suggest large variations in dementia in-hospital mortality that can be associated with both patient-level and hospital-level factors. Targeted and coordinated dementia care should be promoted to reduce in-hospital mortality and provide more equitable and quality dementia care.

